# Arterial stiffness and cardiometabolic health in omnivores and vegetarians: a cross-sectional pilot study

**DOI:** 10.1186/s13104-022-05957-w

**Published:** 2022-02-19

**Authors:** Selicia T. Mayra, Carol S. Johnston

**Affiliations:** 1 Department of Nutrition and Health Science, Ball State University, 1613 W Riverside Ave, Muncie, IN 47303 USA; 2 College of Health Solutions, Arizona State University, 550 N. 3rd Street, Phoenix, AZ 85004 USA

**Keywords:** Arterial stiffness, Vegetarian eating patterns, Aging, Plant-based diets, Nutrition, Cardiometabolic health

## Abstract

**Objective:**

Arterial stiffness is a strong predictor of cardiovascular mortality, and often precedes elevations in blood pressure. This cross-sectional pilot study examined differences in arterial stiffness, blood pressure, cardiometabolic markers, anthropometric outcomes, and inflammation in vegetarians and matched omnivores. Participants were healthy, non-smoking adults (18–65 years old) adhering to either a vegetarian/vegan or omnivore diet. Omnivores were matched to vegetarians using broad body mass index (BMI) categories.

**Results:**

Arterial stiffness trended higher in omnivores versus vegetarians (7.0 ± 1.5 and 6.8 ± 1.1 m/s, respectively; p = 0.073). This trend was mainly driven by the male omnivores (p = 0.006 for gender effect and p = 0.294 for eating pattern effect). Omnivores displayed higher HDL concentrations compared to vegetarians, 63.8 ± 18.5 and 55.2 ± 16.9 mg/dL; however, total cholesterol/HDL ratio did not vary significantly between groups; p = 0.310. In men, a vegetarian eating pattern may reduce arterial stiffness; however, this benefit may be limited in women, particularly those who are premenopausal. Future research should examine arterial stiffness and cardiometabolic health outcomes in younger versus older female vegetarians, as these data can provide valuable insights on the role of plant-based eating patterns on arterial stiffness and cardiometabolic health.

## Introduction

Conditions affecting the cardiovascular system are the leading cause of mortality worldwide [1]. In 2019 alone, 17.9 million deaths were attributed to cardiovascular diseases (CVD), representing a global death toll of 32%. Early detection of CVD risk is crucial for disease detection and amelioration. Arterial stiffening (e.g., the loss of elastic compliance in the vascular wall) is a strong predictor of cardiovascular mortality, and often precedes elevations in blood pressure. Aging is closely associated with arterial stiffness, and further evidence suggests that unhealthful lifestyle practices, including high meat, sodium, and alcohol consumption, smoking, and sedentary behaviors also contribute to the progression of arterial stiffness [2–4].

Pulse wave velocity (PWV) represents a non-invasive marker of disease risk, and, to date, carotid-femoral pulse wave velocity (Cf-PWV) is the most studied technique to quantify arterial stiffness in population-based studies [5]. Cf-PWV is the velocity of the arterial pulse as it travels along the vessel wall and is measured from the carotid to the femoral artery [6]. Reference values range from 4.6 to 7.5 m/s for young, healthy adults and increase progressively with age (+ 200% between the second and ninth decade of life) [7, 8]. PWV values > 10 m/s are considered high risk for cardiovascular events.

This cross-sectional study examined associations between arterial stiffness (Cf-PWV), brachial artery and central aortic blood pressures, cardiometabolic markers, and inflammation by eating pattern type (vegetarian versus omnivore). We hypothesized that these biomarkers would be more favorably aligned to a vegetarian eating pattern compared to an omnivore diet.

## Methods

### Study design

Data collection for this cross-sectional study occurred from February to July 2019 and consisted of a single in-person visit to the Healthy Lifestyle research facility located in downtown Phoenix, Arizona, USA. Study outcomes were arterial stiffness (Cf-PWV); blood pressure [systolic blood pressure (SBP), diastolic blood pressure (DBP), central systolic blood pressure (CSBP), and central diastolic blood pressure (CDBP)]; cardiometabolic markers [total cholesterol, HDL and LDL cholesterol, and triglycerides], and inflammation [high-sensitivity C-reactive protein (hs-CRP)]. Anthropometric measures [body weight, body mass index (BMI), and waist and hip circumferences], and physical activity [Godin-Shephard Leisure-Time Physical Activity Questionnaire] [9] were also recorded. To increase the validity of inferences and to reduce bias, the research team, including the research nurse and phlebotomist were blinded to group allocation, and data were analyzed by the principal investigator.

### Participants

Healthy, non-smoking adults (18–65 years old) adhering to either a vegetarian/vegan or omnivore diet were recruited from a campus population. A vegetarian/vegan self-identified as ‘eating no meat, poultry, or fish’ and were distinguished from individuals who reported consuming ‘meat, fish, and/or poultry on occasion but not daily.’ Pregnant or recently pregnant (within the past three months) women, lactating women, individuals with chronic health conditions as well as those taking medication for these conditions, and individuals engaged in > 150 min per week of moderate to vigorous intensity physical activity were excluded from the study. To create comparable groups, omnivores were matched to vegetarians using broad BMI (18.5 to < 25.0, 25.0 to < 30.0, and ≥ 30.0 kg/m^2^) and age (18–25, 30–40, and 50–65 years) categories. Written informed consent was obtained for all participants, and the study was approved by the Institutional Review Board at Arizona State University.

### Study outcomes

#### Arterial stiffness and blood pressure

Participants were instructed to lay supine in a dimly lit, temperature-controlled room for 10 min. A non-invasive SphygmoCor XCEL (AtCor Medical, Sydney, NSW, Australia) system assessed Cf-PWV. A blood pressure cuff was placed around each participant’s upper thigh and brachial artery of the non-dominant arm. Carotid and femoral pulse rates were identified and recorded, and measured distances from the femoral pulse to the topmost point above of the blood pressure cuff, from the sternal notch to the topmost point above the blood pressure cuff, and from the carotid pulse to the sternal notch were entered into the SphygmoCor XCEL software. A tonometer was placed directly above the carotid pulse, and firm yet stable pressure was applied allowing the SphygmoCor XCEL device to compute Cf-PWV. Brachial artery and central aortic blood pressures were also recorded. Measurements were obtained in triplicate, and the mean of the last two measurements was recorded.

#### Cardiometabolic markers and inflammation

One 10 mL fasting (except water for 12 h) blood sample was acquired from each participant’s antecubital vein. Total cholesterol was measured using a cholesterol fluorometric assay kit (Cat. No. 10007640, Cayman Company, Ann Arbor, MI, USA), HDL cholesterol was assessed with a quantitation kit (Cat. No. MAK045-1KT, Millipore-Sigma, Burlington, MA, USA), LDL cholesterol was calculated using the Friedewald formula [10], triglycerides were measured using a Colorimetric Assay Kit (Cat. No. 10010303, Cayman Company, Ann Arbor, MI), and an ELISA kit (Cat. No.10011236) assessed hs-CRP.

#### Anthropometry measurements

Height (cm) was recorded using a stadiometer (SECA 217, Tiger Medical, Irvington, NJ, USA). Weight (kg) was obtained using a research-grade, calibrated total body composition analyzer (Cat. No. TBF-300, Tanita, Arlington Heights, IL, USA). Waist and hip circumferences were obtained with a SECA ergonomic measuring tape (Cat. No. 2011717009, Tiger Medical, Irvington, NJ, USA). For waist circumference, the measuring tape was positioned horizontally around the abdomen at the midpoint between the lowest rib and the top of the iliac crest. For hip circumference, the measuring tape was positioned around the widest portion of the posterior, parallel to the floor. Measurements were obtained twice and averaged.

### Statistical analysis

Power analysis indicated that a sample size of 58 was suitable to detect a 0.6 m/s difference in PWV at a 5% level of significance assuming a standard deviation of 0.8 [11]. Data are represented as mean ± SD. Data that violated the Gaussian distribution were transformed prior to analyses (Cf-PWV, HDL cholesterol, hs-CRP, total cholesterol/HDL ratio, and triglyceride/HDL ratio). Group differences for Cf-PWF, brachial artery and central aortic blood pressures, and cardiometabolic markers were analyzed using univariate analyses controlling for age, adiposity, and gender. Additionally, a multivariate ANOVA was conducted for the cardiometabolic factors that were correlated (SBP, DBP, LDL cholesterol, total cholesterol, and triglycerides), and a two-way ANOVA was utilized to assess Cf-PWV by diet plan and gender. Descriptive data were compared using the Mann–Whitney U test or the Chi-Square test. Spearman coefficients were computed to compare relationships between variables. All analyses were performed using the Statistical Package for the Social Sciences, version 27 (IBM Corp., Armonk, NY, USA), and statistical significance was established at a p-value ≤ 0.05.

## Results

### Participants

Of 149 eligible respondents, 55 individuals agreed to enroll in the study and completed the in-person visit (22 omnivores and 33 vegetarians). Thirteen vegetarians self-reported occasional meat consumption and were excluded from analyses. The median duration of adherence to a vegetarian eating pattern was nine years (range: 2–42 years). Participants were 75% Caucasian. Participant characteristics are displayed in Table 1; adiposity measures did differ by eating pattern. Gender, age, and BMI were significantly related to Cf-PWV, and these variables were controlled in the outcome analyses.

**Table 1:** Participant characteristics by eating pattern type*

	Omnivores	Vegetarians	p value
Gender (M/F)	5/17	4/16	0.830
Age (y)	37.4 ± 15.4	34.2 ± 12.5	0.623
Body weight (kg)	69.1 ± 15.2	64.2 ± 9.4	0.358
BMI (kg/m^2^)	24.4 ± 3.9	22.3 ± 2.8	0.030
Waist circumference (cm)	76.2 ± 11.7	72.2 ± 8.4	0.242
Hip circumference (cm)	100.8 ± 8.2	95.8 ± 6.1	0.030
Physical activity score	46.2 ± 22.5	58.2 ± 54.3	0.920

*Data are mean ± SD; p-values represent Mann–Whitney U test or Chi-Square test for gender

### Comparison of study outcomes by eating pattern type

**Fig. 1:**
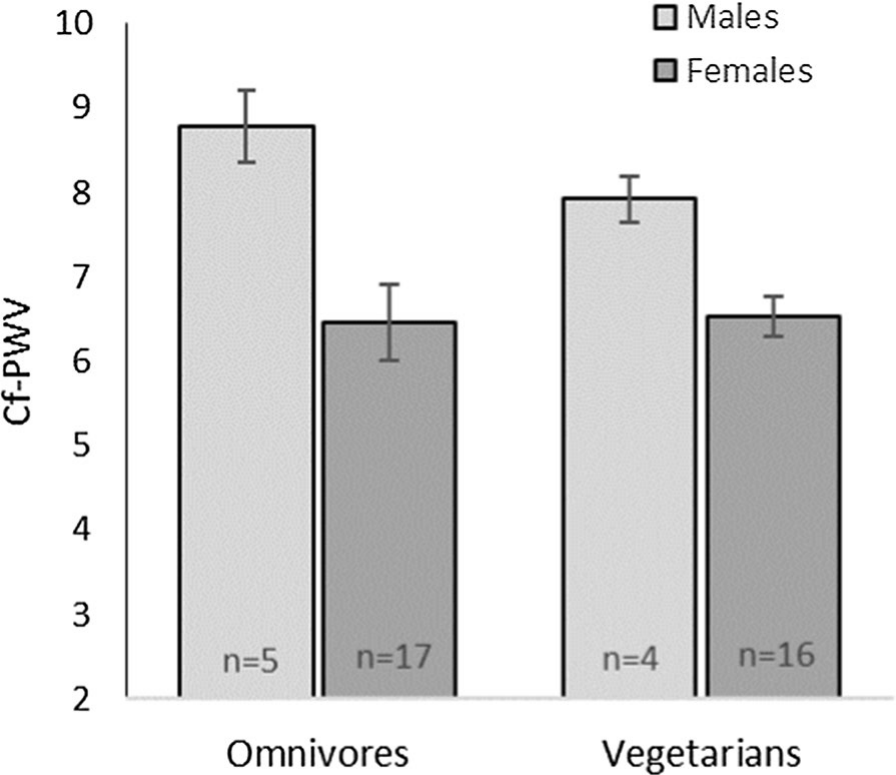
Carotid-femoral pulse wave velocity (Cf-PWV) by gender and eating pattern type. (p = 0.006 for gender effect and p = 0.294 for eating pattern effect; two-way ANOVA controlling for age and adiposity)

Cf-PWV trended higher in the omnivores compared to the vegetarians (7.0 ± 1.5 and 6.8 ± 1.1 m/s, respectively; p = 0.073) (shown in Table 2). Although, when separated by gender, the trend by eating pattern was mainly driven by the male participants; p = 0.006 for gender effect and p = 0.294 for eating pattern effect (shown in Fig. 1).Cf-PWV values were 8.8 ± 1.0 and 7.9 ± 0.9 m/s for the male omnivores and vegetarians compared to 6.5 ± 1.1 and 6.5 ± 0.9 m/s for the female omnivores and vegetarians, respectively.

**Table 2:** Outcome variables by eating pattern type*

	Omnivores	Vegetarians	p value
Cf-PWV (m/s)	7.0 ± 1.5	6.8 ± 1.1	0.073
SBP (mmHg)	119.8 ± 13.2	115.8 ± 15.6	0.891
DBP (mmHg)	73.9 ± 8.2	71.6 ± 11.4	0.998
CSBP (mmHg)	107.9 ± 13.2	103.5 ± 15.2	0.975
CDBP (mmHg)	73.9 ± 7.9	72.3 ± 11.5	0.878
Total cholesterol (mg/dL)	203.4 ± 39.7	179.9 ± 39.1	0.119
HDL cholesterol (mg/dL)	63.8 ± 18.5	55.2 ± 16.9	0.013
Total cholesterol/HDL ratio	3.38 ± 1.0	3.46 ± 1.0	0.310
LDL cholesterol (mg/dL)	122.2 ± 38.6	105.1 ± 32.1	0.255
Triglycerides (mg/dL)	86.7 ± 21.1	97.9 ± 41.8	0.212
Triglyceride/HDL ratio	2.1 ± 0.7	1.6 ± 0.9	0.075
hs-CRP (mg/L)	1.5 ± 2.2	2.0 ± 2.4	0.459

*Data are mean ± SD; p-values represent univariate analysis controlling for age, adiposity, and gender

Blood pressure did not differ significantly between the omnivores and vegetarians (SBP = 119.8 ± 13.2 and 115.8 ± 15.6 mmHg; DBP = 73.9 ± 8.2 and 71.6 ± 11.4 mmHg; CSBP 107.9 ± 13.2 and 103.5 ± 15.2 mmHg; and CDBP 73.9 ± 7.9 and 72.3 ± 11.5 mmHg). HDL cholesterol differed significantly between groups with omnivores displaying higher HDL concentrations than vegetarians; 63.8 ± 18.5 and 55.2 ± 16.9 mg/dL. Other lipid values (p > 0.05), total cholesterol/HDL ratio (3.38 ± 1.0 and 3.46 ± 1.0, for the omnivores and vegetarians, respectively; p = 0.310), and hs-CRP (1.5 ± 2.2 and 2.0 ± 2.4 mg/L, respectively; p = 0.541) did not differ between groups. Since the cardiometabolic factors were closely related, a multivariate ANOVA was conducted controlling for age, adiposity, and gender. There was no statistically significant difference between the diet groups on the combined dependent variables (LDL cholesterol, SBP, DBP, total cholesterol, triglycerides; p = 0.269).

## Discussion

Cf-PWV is the gold-standard, non-invasive measure of arterial stiffness, and it is considered a reliable indicator of CVD risk [12, 13]. Although this study was not conducted to compare Cf-PWV by gender and eating pattern, and only 20% of the sample was male, these preliminary data suggest that a vegetarian eating pattern may be particularly advantageous for reducing CVD risk in young men. The data demonstrated a gender effect on Cf-PWV; however, the small portion of men in the sample did not permit adequate statistical power to demonstrate a significant gender by eating pattern effect.

Age is a strong predictor of arterial stiffness, and PWV increases 6–8% with each decade of life after age 50 [14]. The lower arterial stiffness for women versus age-matched men is noted mainly in the decades prior to, but not after, menopause [7]. Age ranged from 18 to 64 years in the current study (mean: 35.8 ± 14 years), with a similar age span by eating pattern by design. Hence, the gender effect noted herein may have been more pronounced in an exclusively young sample. Estrogen likely mediates this gender effect as many of its functions promote cardiovascular health, including the stimulation of nitric oxide, a potent vasodilator, the alleviation of oxidative stress, and the attenuation of salt sensitivity [15–18]. To our knowledge, the only other investigation comparing PWV in vegetarians and omnivores was a 2016 investigation that reported significantly lower PWV (− 8%) in healthy male vegetarians (n = 44) versus a comparable omnivore control sample (n = 44; 7.1 ± 0.8 and 7.7 ± 0.9 m/s, respectively) [11]. These data suggest that in men, adoption of a vegetarian eating pattern may reduce CVD risk by improving PWV; however, this benefit may be limited in women, particularly those who are premenopausal. Future research examining PWV and CVD risk in younger versus older women who adhere to vegetarian eating as well as men across the lifespan would provide much-needed information on the benefits of meatless eating patterns to improve PWV and reduce CVD risk.

The difference in mean Cf-PWV between the two eating patterns was small (0.2 m/s or 3%); however, this difference is similar to reductions noted for PWV in controlled feeding trials. In a recent meta-analysis of 12 randomized controlled trials (n = 1007 participants), Chu et al. observed 0.148 m/s mean reduction in PWV in participants receiving fish oil supplements compared to controls (p = 0.013) [19]. Trial durations averaged 14 weeks, and participant PWV at baseline averaged ~ 8 m/s. A meta-analysis that examined the impact of soy isoflavone supplementation on PWV (four qualifying controlled, randomized trials encompassing 441 participants) demonstrated a significant reduction in average PWV (− 0.33 m/s; p = 0.02) [20]. Trial durations ranged from 4 to 12 weeks, and baseline PWV averaged ~ 9 m/s. These investigations suggest that a 2–3% reduction in PWV is noteworthy. Moreover, a 0.2 m/s reduction in PWV is meaningful considering the age-associated rise in PWV beginning at age 50: 0.43–0.93 m/s per decade in normal and hypertensive populations, respectively [21].

## Limitations/conclusions

This pilot study was limited by the small sample size and the relatively young age of the majority of female participants. Despite this, this is the first known examination of arterial stiffness in female vegetarians in comparison to matched omnivores, and the data can inform planning decisions for future observational studies and intervention trials. CVD will impact over one-third of Americans as they age. Diet-based strategies that effectively reduce CVD risk in men and women throughout the life cycle are warranted. It will also be prudent to address how smoking status might influence the progression of arterial stiffness in vegetarian populations.

## Data Availability

Requests for data described in the manuscript may be made to the corresponding author.

## Abbreviations

PWV: Pulse wave velocity
Cf-PWV: Carotid-femoral pulse wave velocity
SBP: Systolic blood pressure
DBP: Diastolic blood pressure
CSBP: Central systolic blood pressure
CDBP: Central diastolic blood
HDL: High density lipoprotein
LDL: Low density lipoprotein
hs-CRP: High-sensitivity C-reactive protein
